# Development and validation of an interpretable machine learning model for venous thromboembolism risk prediction in patients with lung cancer: a real-world study

**DOI:** 10.3389/fmed.2026.1853920

**Published:** 2026-07-08

**Authors:** Ao Xia, Jingyuan Liu, Juanjuan Song, Yu Han, Bingjie Ding, Ao Xie, Chang Liu, Xiance Tang, Wenqun Xing, Dongmin Zhou, Liu Liu, Hu Zhou

**Affiliations:** 1 Department of Critical Care Medicine, The Affiliated Cancer Hospital of Zhengzhou University & Henan Cancer Hospital, Zhengzhou, China; 2 Department of Hematology, The Affiliated Cancer Hospital of Zhengzhou University & Henan Cancer Hospital, Hemostasis and Thrombosis Diagnostic Engineering Research Center of Henan Province, Zhengzhou, China; 3 Department of Hematology, Huaihe Hospital of Henan University, Kaifeng, China; 4 Department of Follow-up Center, The Affiliated Cancer Hospital of Zhengzhou University & Henan Cancer Hospital, Zhengzhou, China; 5 Department of Thoracic Surgery, The Affiliated Cancer Hospital of Zhengzhou University & Henan Cancer Hospital, Zhengzhou, Henan, China; 6 Department of Hematology, The First Affiliated Hospital of Zhengzhou University, Zhengzhou, China

**Keywords:** lung cancer, machine learning, risk prediction, SHapley Additive exPlanation, venous thromboembolism

## Abstract

**Background:**

Venous thromboembolism (VTE) is a common complication in patients with lung cancer and remains difficult to predict accurately using existing risk assessment tools. This study aimed to develop and validate a machine learning model for individualized prediction of VTE risk in patients with lung cancer.

**Methods:**

A total of 7,959 patients with lung cancer from the Affiliated Cancer Hospital of Zhengzhou University and Henan Cancer Hospital were included, among whom 1,333 developed VTE. Patients were randomly assigned to the training and testing cohorts in a 7:3 ratio. Candidate predictors were identified from 67 clinical variables using multivariable logistic regression and least absolute shrinkage and selection operator (LASSO) regression. Seven prediction models were developed based on the selected variables, including logistic regression, Decision Tree, Random Forest, Extreme Gradient Boosting, Light Gradient Boosting Machine, Support Vector Machine, and Artificial Neural Network (ANN). Model performance was evaluated using the area under the receiver operating characteristic curve, F1 score, calibration curves, and decision curve analysis, and SHapley Additive exPlanation was used to interpret feature contributions.

**Results:**

Twelve variables were selected for model construction: sex, age, anticoagulant use, atherosclerosis, chemotherapy, intermittent pneumatic compression, radiotherapy, CYFRA21-1, NSE, central venous catheter placement, thrombin time, and D-dimer. Among the seven models, ANN showed the best overall performance, with the highest mean cross-validated area under the receiver operating characteristic curve in the training cohort (0.825 ± 0.016), a testing-cohort value of 0.817 (95% CI 0.794–0.840), and the highest F1 score. Calibration analysis showed good agreement between predicted and observed probabilities, and decision curve analysis supported the potential clinical utility of the model across relevant threshold probabilities. SHapley Additive exPlanation further illustrated the contribution and relative importance of each predictor.

**Conclusion:**

An interpretable ANN model was developed for individualized prediction of VTE risk in patients with lung cancer. The model incorporates 12 routinely available clinical variables and may support identification of patients at high risk of VTE, risk stratification, and clinical decision-making.

## 1 Introduction

Venous thromboembolism (VTE), including deep venous thrombosis and pulmonary embolism, is associated with adverse outcomes. Compared with the general population, patients with cancer have a markedly increased risk of VTE (1). Thromboembolism is also a leading cause of death in patients receiving outpatient chemotherapy (2). In an autopsy-based cohort study, pulmonary embolism was identified in 12% of patients with cancer, and two-thirds of these events were considered fatal (3). In addition, both short-term and long-term mortality rates are substantially higher in patients with cancer-associated venous thrombosis than in those without this complication (4, 5). Certain malignancies, particularly pancreatic cancer and lung cancer, are associated with especially poor outcomes when complicated by VTE (4).

Beyond its effect on mortality, cancer-associated VTE (CAT) has a substantial impact on morbidity and clinical management. CAT is associated with increased hospitalization, interruption or delay of anticancer therapy, psychological burden, and higher healthcare costs (6–8). VTE-related complications may also necessitate modifications to treatment protocols, further complicating patient care (6). Early identification of patients at high risk of VTE is therefore important for risk stratification and targeted thromboprophylaxis.

Several risk assessment tools for CAT have been proposed (9, 10). The Khorana score (9) remains the most widely implemented risk assessment tool for patients scheduled to receive chemotherapy, but its discriminatory performance in patients with lung cancer is limited (11). Although the COMPASS-CAT score has shown improved performance, further validation is still needed. These limitations highlight the need for a robust risk assessment tool specifically tailored to patients with lung cancer.

In this study, we aimed to identify risk factors for VTE in patients with lung cancer and to develop a prediction model for individualized risk assessment using routinely available clinical variables.

## 2 Materials and methods

### 2.1 Patient population

Comprehensive data were extracted from electronic medical records (EMRs) of patients treated at the Affiliated Cancer Hospital of Zhengzhou University & Henan Cancer Hospital. We systematically collected demographic characteristics, clinical data and laboratory indicators for each eligible patient. The inclusion criteria comprised: (1) age ≥ 18 years; (2) at least once hospitalization episode; (3) newly histologically confirmed lung carcinoma; and (4) hospitalization between January 2017 and March 2024. The exclusive criteria was as follows: (1) incomplete medical records, laboratory data, or other necessary clinical information; (2) concurrent diagnosis of other malignant and; (3) follow-up duration<6 months.

### 2.2 Outcome definition

The primary outcome was any objectively confirmed VTE occurring within 180 days after the index admission. The index admission was defined as the first hospitalization at our institution during the study period. Follow-up was uniformly defined as the period from the date of index admission to 180 days thereafter for all patients. Patients were considered to have reached the outcome if VTE was diagnosed either during the index hospitalization or during any subsequent readmission or outpatient follow-up visit at our institution within 180 days after the index admission. Deep vein thrombosis was diagnosed by venous ultrasonography, and pulmonary embolism was diagnosed by computed tomography pulmonary angiography or pulmonary angiography. Only imaging-confirmed events documented in the medical records were considered outcome events. All diagnoses were independently verified by at least two experienced radiologists.

### 2.3 Selection of clinical variables associated with VTE development

Initially, we performed manual feature selection based on clinical expertise, previous literature evidence, and data accessibility (12, 13). From hospital EMRs, we extracted the following comprehensive information categories. (1) General data, including sex, age, body mass index, Eastern Cooperative Oncology Group performance status, pathology, TNM staging; (2) treatment- and procedure-related variables including anticoagulants, antiplatelet drugs, hemostatic drugs, vasopressors, chemotherapy drugs, radiotherapy, lung cancer surgery, intermittent pneumatic compression (IPC), central venous catheter (CVC) placement; (3) comorbidities, including hypertension, diabetes, cerebral infarction, cerebral hemorrhage, coronary heart disease, heart failure, chronic obstructive pulmonary disease; and (4) Laboratory indicators, including complete blood count, liver function, kidney function, tumor markers and coagulation function.

The timing of predictor collection was defined according to variable type. Baseline demographic, clinical, comorbidity, and laboratory variables were collected at the index admission, and laboratory indicators were based on the first blood test performed during that admission. In contrast, treatment- and procedure-related variables were not considered purely baseline variables but were defined as pre-event treatment/procedure exposures. For patients who developed VTE, these variables were coded only if documented before the date of VTE diagnosis; information recorded after VTE occurrence was not used for model development. For patients without VTE, these variables were defined using records available before the end of the 180-day observation period. Anticoagulant use was defined as pre-VTE anticoagulant-related exposure documented in the medical records, rather than as a strictly defined pharmacologic VTE chemoprophylaxis variable.

Therefore, the model should be interpreted as a pre-event clinical prediction model for 180-day VTE risk, incorporating baseline admission information and pre-VTE treatment/procedure exposures, rather than as a model based exclusively on baseline admission variables or a discharge-only model.

### 2.4 Data preprocessing

The participants were divided into a training cohort and a testing cohort at a ratio of 7:3 by stratified random sampling according to the outcome variable. The training cohort was used to develop the models, and the testing cohort was used for internal validation.

Clinical data underwent preprocessing before model development, including outlier handling and assessment of missing values. Variables with a missing rate greater than 20% were generally excluded from subsequent analyses to reduce potential bias, although clinically important variables could be retained after comprehensive evaluation (14). For the remaining continuous and categorical variables with missingness of 20% or less, multiple imputation by chained equations was performed separately in the training and testing cohorts to avoid information leakage. Binary variables were coded as 0/1 before model development. For comorbidities, the presence of the condition was coded as 1 and its absence as 0. Sex was coded as male = 1 and female = 0. In this dataset, the absence of a recorded diagnosis was considered as absence of the condition, since positive findings are systematically documented (15). Therefore, these variables had no missing values and did not require imputation.

### 2.5 Model construction and validation

For model construction, candidate predictors were identified in the training cohort using two complementary approaches: multivariable logistic regression and LASSO regression (14). In the logistic regression analysis, variables with *p* < 0.05 in the univariate analysis were entered into the multivariable model, and those that remained significant were retained. LASSO regression was used to select informative predictors through L1-penalised shrinkage, thereby reducing predictor redundancy and limiting model complexity. Predictors consistently identified by both methods were considered more robust and were included in subsequent model development. Clinical relevance and interpretability were also considered in the final selection of predictors.

The selected features were subsequently used to develop seven prediction models: logistic regression (LR), Decision Tree (DT), Random Forest (RF), Extreme Gradient Boosting (XGBoost), Light Gradient Boosting Machine (LightGBM), Support Vector Machine (SVM), and Artificial Neural Network (ANN). LR was used as a conventional linear baseline model, and the other six were machine learning (ML) models. To reduce the risk of overfitting and enhance model stability, hyperparameters were tuned on the training dataset using grid search with five-fold cross-validation to enhance model stability and generalizability.

Model performance was evaluated in terms of discrimination, calibration, and clinical utility. Discrimination was assessed using the area under the receiver operating characteristic curve (AUROC), accuracy, precision, sensitivity, specificity, positive predictive value and negative predictive value. AUROC was used to assess overall discriminative ability, whereas the F1 score, defined as the harmonic mean of precision and recall, was used as an important complementary criterion for model selection (16). Because VTE is a relatively infrequent but clinically important outcome, the F1 score was considered useful for evaluating the balance between identifying true high-risk patients and limiting false-positive classification. Among models with similar AUROC values, the model with the highest F1 score was favored. Youden’s J statistic was additionally calculated as a summary measure of diagnostic effectiveness. Calibration was examined using calibration curves to assess the agreement between predicted probabilities and observed outcomes. Clinical utility was evaluated using decision curve analysis (DCA) across a range of threshold probabilities. The final model was selected by considering five-fold cross-validation performance, test cohort performance, and the consistency between training and test results.

To further evaluate the added value of the ANN model, we performed a *post hoc* benchmark comparison with the Khorana score in the test cohort. Because BMI was required for calculation of the original Khorana score, the primary comparison was performed in the BMI-complete test subset. AUCs with 95% confidence intervals were calculated, and paired AUCs were compared using the DeLong test. Given the high missing rate of BMI, a sensitivity analysis was also performed using a BMI-excluded Khorana score, calculated by summing all original Khorana components except the BMI criterion. This BMI-excluded score was used only as an exploratory sensitivity analysis and not as a validated replacement for the original Khorana score.

### 2.6 Model explanation

To enhance interpretability, SHapley Additive exPlanations (SHAP) were applied to quantify the contribution of each feature to model predictions. SHAP values were calculated for individual predictions, allowing estimation of the direction and magnitude of each feature’s effect while accounting for interactions among features. Positive SHAP values indicated that a feature increased the predicted risk, negative values indicated a decrease, and values close to zero suggested minimal influence (17).

### 2.7 Statistical analysis

Continuous variables conforming to a normal distribution were summarized as mean ± standard deviation, whereas skewed continuous variables were reported as median with interquartile range (Q1–Q3). Categorical variables were expressed as counts and percentages. Baseline characteristics between the training and test cohorts were described descriptively without formal statistical testing, as such comparisons do not influence model development.

**Figure 1:**
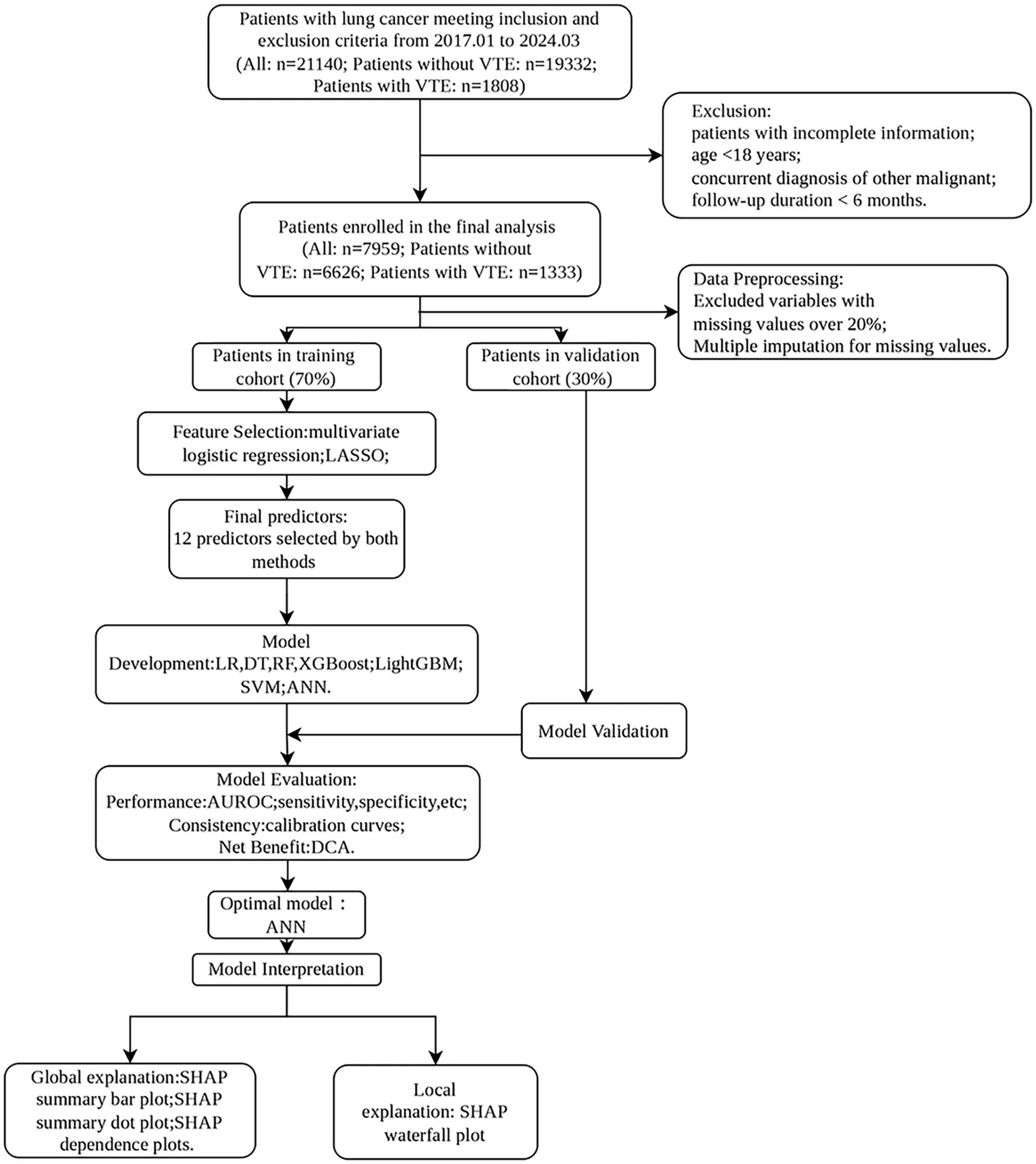
Overall methodological workflow of the study.

Baseline characteristics and feature selection were analyzed using R (version 4.4.2). Prediction model development and evaluation, hyperparameter tuning, and SHAP-based model interpretation were performed using Python (version 3.10). All statistical tests were two-sided, and *p* < 0.05 was considered statistically significant. The overall methodological workflow is detailed in Figure 1.

### 2.8 Ethics statement

The study protocol received ethical approval from the Medical Ethics Committee of Affiliated Cancer Hospital of Zhengzhou University & Henan Cancer Hospital (approval 2025–282-001), with approval valid for one year. It was registered in the Medical Research Registration Information System under registration number MR-41-26-012774. The requirement for informed consent was waived due to the retrospective nature of the study. This study complied with the principles of the Declaration of Helsinki. All data were de-identified before analysis and are reported in aggregate.

## 3 Results

### 3.1 Patient characteristics

A total of 21,140 patients with lung cancer were initially screened. After application of the predefined eligibility criteria, 7,959 patients were included in the final analysis. Before model development, clinical data underwent preprocessing, including outlier handling and assessment of missing values. Variables with more than 20% missingness were excluded to reduce potential bias, including body mass index, Eastern Cooperative Oncology Group performance status, C-reactive protein, and TNM stage. D-dimer was retained despite a missing rate of 20% because of its established role in VTE risk assessment. The missing rates of all candidate variables are summarized in Supplementary Table 1. The median age of the study population was 61 years, and 63.01% were male. During follow-up, 1,333 patients developed VTE, corresponding to an incidence of 16.7%.

The study population was randomly divided into a training cohort (*n* = 5,572) and a testing cohort (*n* = 2,387) at a 7:3 ratio. Outcome-stratified sampling was used to ensure a balanced distribution of VTE events between the two cohorts. Baseline characteristics of the training and testing cohorts are summarized in Table 1. No significant differences were observed between the cohorts in demographic, clinical, or laboratory variables, including age, sex, tumor type, D-dimer levels, and anticoagulant use (all *p* > 0.05). These findings indicate that the cohorts were well balanced and suitable for model development and validation. Baseline characteristics of the overall cohort stratified by VTE status are presented in Table 2. Patients who developed VTE tended to be older, more frequently male, and had higher D-dimer levels than those without VTE. These results highlight key clinical features associated with VTE and confirm that the training and testing cohorts were representative of the overall study population. This provides a robust foundation for predictive modeling.

**Table 1:** Baseline characteristics of the training and testing set.

Characteristics	All (*N* = 7,959)	Testing (*N* = 2,387)	Training (*N* = 5,572)	*p*-value
Sex				0.366
Female	2942 (36.96%)	864 (36.20%)	2078 (37.29%)	
Male	5017 (63.04%)	1523 (63.80%)	3494 (62.71%)	
Age (year)	61.00[53.00, 67.00]	61.00[53.00, 67.00]	61.00[53.00, 67.00]	0.278
Operation				0.470
No	7260 (91.22%)	2169 (90.87%)	5091 (91.37%)	
Yes	699 (8.78%)	218 (9.13%)	481 (8.63%)	
Pathology				0.860
NSCLC	6221 (78.16%)	1867 (78.22%)	4354 (78.14%)	
SCLC	1532 (19.25%)	455 (19.06%)	1077 (19.33%)	
Undifferentiated	206 (2.59%)	65 (2.72%)	141 (2.53%)	
Hypertension				0.246
No	5863 (73.67%)	1737 (72.77%)	4126 (74.05%)	
Yes	2096 (26.33%)	650 (27.23%)	1446 (25.95%)	
Diabetes				0.165
No	7083 (88.99%)	2106 (88.23%)	4977 (89.32%)	
Yes	876 (11.01%)	281 (11.77%)	595 (10.68%)	
Cerebral infarction				1.000
No	7298 (91.69%)	2189 (91.71%)	5109 (91.69%)	
Yes	661 (8.31%)	198 (8.29%)	463 (8.31%)	
Cerebral hemorrhage				0.098
No	7917 (99.47%)	2369 (99.25%)	5548 (99.57%)	
Yes	42 (0.53%)	18 (0.75%)	24 (0.43%)	
Hemostatic drugs				0.905
No	7906 (99.33%)	2372 (99.37%)	5534 (99.32%)	
Yes	53 (0.67%)	15 (0.63%)	38 (0.68%)	
Coronary heart disease				0.723
No	7506 (94.31%)	2255 (94.47%)	5251 (94.24%)	
Yes	453 (5.69%)	132 (5.53%)	321 (5.76%)	
HF				0.587
No	7645 (96.05%)	2288 (95.85%)	5357 (96.14%)	
Yes	314 (3.95%)	99 (4.15%)	215 (3.86%)	
COPD				0.566
No	7922 (99.54%)	2378 (99.62%)	5544 (99.50%)	
Yes	37 (0.46%)	9 (0.38%)	28 (0.50%)	
Anticoagulant				1.000
No	4326 (54.35%)	1297 (54.34%)	3029 (54.36%)	
Yes	3633 (45.65%)	1090 (45.66%)	2543 (45.64%)	
Atherosclerosis:				0.465
No	3793 (47.66%)	1153 (48.30%)	2640 (47.38%)	
Yes	4166 (52.34%)	1234 (51.70%)	2932 (52.62%)	
Chemotherapy drugs				1.000
No	1747 (21.95%)	524 (21.95%)	1223 (21.95%)	
Yes	6212 (78.05%)	1863 (78.05%)	4349 (78.05%)	
IPC				0.566
No	4364 (54.83%)	1321 (55.34%)	3043 (54.61%)	
Yes	3595 (45.17%)	1066 (44.66%)	2529 (45.39%)	
Vasopressors				0.881
No	5327 (66.93%)	1601 (67.07%)	3726 (66.87%)	
Yes	2632 (33.07%)	786 (32.93%)	1846 (33.13%)	
Radiotherapy				0.450
No	4980 (62.57%)	1509 (63.22%)	3471 (62.29%)	
Yes	2979 (37.43%)	878 (36.78%)	2101 (37.71%)	
CVC placement				0.992
No	1975 (24.81%)	593 (24.84%)	1382 (24.80%)	
Yes	5984 (75.19%)	1794 (75.16%)	4190 (75.20%)	
WBC (10^9^/L)	6.98 [5.64; 8.71]	6.97 [5.66; 8.77]	6.98 [5.63; 8.69]	0.864
RBC (10^12^/L)	4.44 [4.09; 4.78]	4.44 [4.08; 4.78]	4.44 [4.10; 4.78]	0.824
HGB (g/L)	134.00 [123.00; 145.00]	134.00 [123.00; 145.00]	134.00 [123.00; 145.00]	0.940
PLT (10^9^/L)	252.00 [204.00; 309.00]	250.00 [205.00; 308.00]	252.00 [204.00; 309.00]	0.922
NEUT (%)	66.40 [59.70; 72.70]	66.30 [59.50; 72.80]	66.40 [59.80; 72.70]	0.642
LYMPH (%)	25.20 [19.20; 31.60]	25.20 [19.10; 31.80]	25.20 [19.30; 31.60]	0.901
MONO (%)	5.00 [4.20; 6.00]	5.00 [4.20; 6.00]	5.00 [4.20; 6.00]	0.521
EOS (%)	1.90 [1.10; 3.20]	2.00 [1.10; 3.30]	1.90 [1.10; 3.20]	0.241
BASO (%)	0.50 [0.30; 0.70]	0.50 [0.30; 0.70]	0.50 [0.30; 0.70]	0.131
NEUT (10^9^/L)	4.56 [3.48; 6.05]	4.53 [3.45; 6.13]	4.57 [3.49; 6.02]	0.930
LYMPH (10^9^/L)	1.73 [1.35; 2.16]	1.73 [1.35; 2.14]	1.73 [1.35; 2.17]	0.929
MONO (10^9^/L)	0.35 [0.27; 0.46]	0.35 [0.27; 0.46]	0.35 [0.27; 0.46]	0.625
EOS (10^9^/L)	0.13 [0.07; 0.22]	0.13 [0.07; 0.23]	0.13 [0.07; 0.22]	0.324
BASO (10^9^/L)	0.03 [0.02; 0.05]	0.03 [0.02; 0.05]	0.03 [0.02; 0.05]	0.217
HCT (%)	41.00 [37.90; 44.00]	41.00 [37.80; 44.20]	41.00 [37.90; 44.00]	0.590
MCV (fL)	92.50 [89.40; 95.60]	92.60 [89.50; 95.60]	92.50 [89.30; 95.60]	0.439
MCH (pg)	30.40 [29.20; 31.50]	30.40 [29.20; 31.40]	30.40 [29.20; 31.50]	0.709
MCHC (g/L)	327.00 [319.00; 335.00]	327.00 [319.00; 335.00]	327.00 [319.00; 336.00]	0.136
RDW_SD (fL)	43.00 [41.00; 46.00]	43.00 [41.00; 46.00]	43.00 [41.00; 46.00]	0.991
RDW_CV (%)	13.00 [12.00; 14.00]	13.00 [12.00; 14.00]	13.00 [12.00; 13.00]	0.459
PDW (fL)	11.80 [10.40; 14.00]	11.70 [10.30; 14.10]	11.80 [10.40; 14.00]	0.588
MPV (fL)	10.10 [9.50; 10.90]	10.10 [9.50; 10.90]	10.10 [9.50; 10.90]	0.489
PCT (%)	0.26 [0.21; 0.31]	0.26 [0.21; 0.31]	0.26 [0.21; 0.31]	0.977
P_LCR (%)	25.90 [20.90; 31.90]	25.80 [20.60; 31.95]	25.90 [21.00; 31.90]	0.470
TBIL (umol/L)	9.90 [7.40; 13.10]	9.80 [7.40; 13.00]	9.90 [7.40; 13.10]	0.501
DBIL (umol/L)	3.90 [3.10; 5.10]	3.90 [3.05; 5.00]	3.90 [3.10; 5.10]	0.373
IBIL (umol/L)	5.90 [4.20; 8.10]	5.90 [4.20; 8.00]	5.90 [4.20; 8.10]	0.547
ALT (U/L)	16.00 [11.00; 24.00]	16.00 [11.00; 24.50]	16.00 [11.00; 24.00]	0.605
AST (U/L)	18.00 [14.00; 23.00]	18.00 [14.00; 23.00]	17.00 [14.00; 23.00]	0.592
ALP (U/L)	84.00 [69.00; 104.00]	84.00 [69.00; 104.00]	84.00 [69.00; 104.00]	0.691
GGT (U/L)	24.00 [16.00; 40.00]	24.00 [17.00; 40.00]	24.00 [16.00; 40.00]	0.944
TP (g/L)	68.60 [64.30; 72.70]	68.60 [64.20; 72.60]	68.70 [64.40; 72.70]	0.380
ALB (g/L)	41.40 [38.30; 44.30]	41.40 [38.20; 44.20]	41.50 [38.40; 44.40]	0.211
GLOB (g/L)	27.00 [24.10; 30.10]	27.00 [24.10; 30.30]	26.90 [24.10; 30.10]	0.563
A/G	1.50 [1.30; 1.80]	1.50 [1.30; 1.70]	1.50 [1.30; 1.80]	0.337
BUN (mmol/L)	5.00 [4.10; 6.20]	5.10 [4.10; 6.20]	5.00 [4.10; 6.20]	0.890
CREA (umol/L)	62.00 [53.00; 72.00]	62.00 [53.00; 72.00]	62.00 [53.00; 72.00]	0.799
UA (umol/L)	262.00 [214.00; 314.00]	261.00 [213.00; 314.00]	262.00 [214.00; 315.00]	0.620
CYFRA211 (ng/mL)	4.81 [3.06; 9.49]	4.79 [3.08; 9.57]	4.82 [3.05; 9.44]	0.690
NSE (ng/mL)	17.20 [13.31; 25.54]	17.08 [13.34; 24.85]	17.24 [13.30; 26.04]	0.351
CEA (ng/mL)	5.00 [2.46; 22.48]	5.14 [2.47; 22.57]	4.94 [2.45; 22.44]	0.714
PT(s)	11.80 [11.20; 12.60]	11.80 [11.20; 12.60]	11.85 [11.20; 12.60]	0.284
INR	0.97 [0.92; 1.03]	0.98 [0.92; 1.03]	0.97 [0.92; 1.03]	0.178
PT_1 (%)	97.30 [90.20; 104.10]	97.00 [90.20; 104.10]	97.30 [90.69; 104.10]	0.220
APTT(s)	29.00 [25.90; 32.90]	29.10 [26.05; 33.00]	28.90 [25.80; 32.70]	0.076
TT(s)	16.60 [15.70; 17.50]	16.60 [15.60; 17.60]	16.60 [15.70; 17.50]	0.503
FIB (g/L)	3.35 [2.68; 4.18]	3.35 [2.65; 4.18]	3.35 [2.69; 4.19]	0.997
D-Dimer (mg/L)	0.68 [0.40; 1.30]	0.70 [0.39; 1.31]	0.66 [0.40; 1.30]	0.408

NSCLC, non-small cell lung cancer; SCLC, small cell lung cancer; HF, heart failure; COPD, chronic obstructive pulmonary disease; IPC, intermittent pneumatic compression; CVC, central venous catheter; WBC, white blood cell; RBC, red blood cell; Hb, Hemoglobin; PLT, Platelet Count; NEUT%, neutrophil percentage; LYM%, lymphocyte percentage; MONO%, monocyte percentage; ESO%, eosinophil percentage; BASO%, basophil percentage; NEUT, neutrophil count; LYM, lymphocyte count; MONO, monocyte count; ESO, eosinophil count; BASO, basophil count; HCT, hematocrit; MCV, mean corpuscular volume; MCH, mean corpuscular hemoglobin; MCHC, mean corpuscular hemoglobin concentration; RDW-SD, red blood cell distribution width standard deviation; RDW-CV, red blood cell distribution width coefficient of variation; PDW, platelet distribution width; MPV, mean platelet volume; PCT, plateletcrit; LPR, large platelet ratio; TBIL, total bilirubin; DBIL, direct bilirubin; IBIL, indirect bilirubin; ALT, alanine aminotransferase; AST, aspartate aminotransferase; ALP, alkaline phosphatase; GTT, gamma-glutamyl transferase; TP, total protein; ALB, albumin; GLOB, globulins; A/G, albumin/globulin ratio; BUN, blood urea nitrogen; Cr, creatinine; UA, uric acid; CYFRA211, tumor marker CYFRA211; NSE, tumor marker NSE; CEA, carcinoembryonic antigen; PT, prothrombin time; INR, international normalized ratio; PT_1, PT activity; APTT, activated partial thromboplastin time; TT, thrombin time; FIB, fibrinogen.

**Table 2:** Baseline characteristics of subjects stratified by VTE.

Characteristics	All (*N* = 7,959)	No VTE (*N* = 6,626)	VTE (*N* = 1,333)	*p*-value
Sex				<0.001
Female	2942 (36.96%)	2521 (38.05%)	421 (31.58%)	
Male	5017 (63.04%)	4105 (61.95%)	912 (68.42%)	
Age (year)	61.00 [53.00; 67.00]	60.00 [53.00; 67.00]	63.00 [55.00; 69.00]	<0.001
Operation				<0.001
No	7260 (91.22%)	5986 (90.34%)	1274 (95.57%)	
Yes	699 (8.78%)	640 (9.66%)	59 (4.43%)	
Pathology				0.033
NSCLC	6221 (78.16%)	5215 (78.71%)	1006 (75.47%)	
SCLC	1532 (19.25%)	1244 (18.77%)	288 (21.61%)	
Undifferentiated	206 (2.59%)	167 (2.52%)	39 (2.92%)	
Hypertension				0.001
No	5863 (73.67%)	4830 (72.89%)	1033 (77.49%)	
Yes	2096 (26.33%)	1796 (27.11%)	300 (22.51%)	
Diabetes				0.001
No	7083 (88.99%)	5863 (88.48%)	1220 (91.52%)	
Yes	876 (11.01%)	763 (11.52%)	113 (8.48%)	
Cerebral infarction				0.602
No	7298 (91.69%)	6081 (91.77%)	1217 (91.30%)	
Yes	661 (8.31%)	545 (8.23%)	116 (8.70%)	
Cerebral hemorrhage				0.294
No	7917 (99.47%)	6588 (99.43%)	1329 (99.70%)	
Yes	42 (0.53%)	38 (0.57%)	4 (0.30%)	
Hemostatic drugs				0.015
No	7906 (99.33%)	6589 (99.44%)	1317 (98.80%)	
Yes	53 (0.67%)	37 (0.56%)	16 (1.20%)	
Coronary heart disease				0.340
No	7506 (94.31%)	6241 (94.19%)	1265 (94.90%)	
Yes	453 (5.69%)	385 (5.81%)	68 (5.10%)	
HF				0.002
No	7645 (96.05%)	6344 (95.74%)	1301 (97.60%)	
Yes	314 (3.95%)	282 (4.26%)	32 (2.40%)	
COPD				0.894
No	7922 (99.54%)	6596 (99.55%)	1326 (99.47%)	
Yes	37 (0.46%)	30 (0.45%)	7 (0.53%)	
Anticoagulant				<0.001
No	4326 (54.35%)	3382 (51.04%)	944 (70.82%)	
Yes	3633 (45.65%)	3244 (48.96%)	389 (29.18%)	
Atherosclerosis				<0.001
No	3793 (47.66%)	3464 (52.28%)	329 (24.68%)	
Yes	4166 (52.34%)	3162 (47.72%)	1004 (75.32%)	
Chemotherapy drugs				<0.001
No	1747 (21.95%)	1121 (16.92%)	626 (46.96%)	
Yes	6212 (78.05%)	5505 (83.08%)	707 (53.04%)	
IPC				<0.001
No	4364 (54.83%)	3386 (51.10%)	978 (73.37%)	
Yes	3595 (45.17%)	3240 (48.90%)	355 (26.63%)	
Vasopressors				0.001
No	5327 (66.93%)	4383 (66.15%)	944 (70.82%)	
Yes	2632 (33.07%)	2243 (33.85%)	389 (29.18%)	
Radiotherapy				<0.001
No	4980 (62.57%)	3939 (59.45%)	1041 (78.09%)	
Yes	2979 (37.43%)	2687 (40.55%)	292 (21.91%)	
CVC placement				<0.001
No	1975 (24.81%)	1532 (23.12%)	443 (33.23%)	
Yes	5984 (75.19%)	5094 (76.88%)	890 (66.77%)	
WBC (10^9^/L)	6.98 [5.64; 8.71]	6.88 [5.59; 8.50]	7.57 [5.94; 9.66]	<0.001
RBC (10^12^/L)	4.44 [4.09; 4.78]	4.45 [4.11; 4.79]	4.36 [3.97; 4.69]	<0.001
HGB (g/L)	134.00 [123.00; 145.00]	135.00 [124.00; 146.00]	131.00 [120.00; 142.00]	<0.001
PLT (10^9^/L)	252.00 [204.00; 309.00]	251.00 [205.00; 308.00]	256.00 [200.00; 313.00]	0.938
NEUT (%)	66.40 [59.70; 72.70]	65.90 [59.30; 72.10]	69.30 [61.90; 76.20]	<0.001
LYMPH (%)	25.20 [19.20; 31.60]	25.70 [19.90; 32.00]	22.10 [15.90; 29.20]	<0.001
MONO (%)	5.00 [4.20; 6.00]	4.90 [4.10; 5.90]	5.20 [4.20; 6.20]	<0.001
EOS (%)	1.90 [1.10; 3.20]	1.90 [1.10; 3.20]	1.90 [0.90; 3.20]	0.008
BASO (%)	0.50 [0.30; 0.70]	0.50 [0.30; 0.70]	0.40 [0.30; 0.60]	<0.001
NEUT (10^9^/L)	4.56 [3.48; 6.05]	4.47 [3.43; 5.87]	5.16 [3.85; 7.03]	<0.001
LYMPH (10^9^/L)	1.73 [1.35; 2.16]	1.74 [1.37; 2.17]	1.63 [1.25; 2.11]	<0.001
MONO (10^9^/L)	0.35 [0.27; 0.46]	0.34 [0.26; 0.45]	0.39 [0.29; 0.52]	<0.001
EOS (10^9^/L)	0.13 [0.07; 0.22]	0.13 [0.07; 0.22]	0.14 [0.07; 0.23]	0.766
BASO (10^9^/L)	0.03 [0.02; 0.05]	0.03 [0.02; 0.05]	0.03 [0.02; 0.05]	0.892
HCT (%)	41.00 [37.90; 44.00]	41.20 [38.10; 44.20]	40.20 [36.60; 43.30]	<0.001
MCV (fL)	92.50 [89.40; 95.60]	92.60 [89.40; 95.70]	92.20 [89.20; 95.40]	0.092
MCH (pg)	30.40 [29.20; 31.50]	30.40 [29.20; 31.50]	30.30 [29.00; 31.40]	0.017
MCHC (g/L)	327.00 [319.00; 335.00]	327.00 [319.00; 336.00]	327.00 [318.00; 335.00]	0.064
RDW_SD (fL)	43.00 [41.00; 46.00]	43.00 [41.00; 46.00]	44.00 [42.00; 47.00]	<0.001
RDW_CV (%)	13.00 [12.00; 14.00]	13.00 [12.00; 13.00]	13.00 [13.00; 14.00]	<0.001
PDW (fL)	11.80 [10.40; 14.00]	11.80 [10.40; 13.90]	11.60 [10.10; 14.40]	0.020
MPV (fL)	10.10 [9.50; 10.90]	10.20 [9.60; 10.90]	9.90 [9.40; 10.70]	<0.001
PCT (%)	0.26 [0.21; 0.31]	0.26 [0.21; 0.31]	0.25 [0.20; 0.31]	0.026
P_LCR (%)	25.90 [20.90; 31.90]	26.10 [21.10; 32.20]	24.60 [19.80; 30.60]	<0.001
TBIL (umol/L)	9.90 [7.40; 13.10]	9.80 [7.40; 13.00]	10.30 [7.70; 13.70]	0.001
DBIL (umol/L)	3.90 [3.10; 5.10]	3.90 [3.00; 5.00]	4.10 [3.20; 5.40]	<0.001
IBIL (umol/L)	5.90 [4.20; 8.10]	5.90 [4.20; 8.00]	6.00 [4.30; 8.20]	0.113
ALT (U/L)	16.00 [11.00; 24.00]	16.00 [11.00; 24.00]	16.00 [11.00; 26.00]	0.269
AST (U/L)	18.00 [14.00; 23.00]	17.00 [14.00; 23.00]	18.00 [14.00; 25.00]	0.015
ALP (U/L)	84.00 [69.00; 104.00]	84.00 [69.00; 103.00]	86.00 [72.00; 110.00]	<0.001
GGT (U/L)	24.00 [16.00; 40.00]	24.00 [16.00; 39.00]	28.00 [18.00; 49.00]	<0.001
TP (g/L)	68.60 [64.30; 72.70]	68.80 [64.50; 72.80]	67.60 [63.30; 72.30]	<0.001
ALB (g/L)	41.40 [38.30; 44.30]	41.70 [38.62; 44.60]	39.90 [36.70; 42.90]	<0.001
GLOB (g/L)	27.00 [24.10; 30.10]	26.90 [24.00; 30.00]	27.60 [24.50; 31.00]	<0.001
A/G	1.50 [1.30; 1.80]	1.60 [1.40; 1.80]	1.40 [1.20; 1.70]	<0.001
BUN (mmol/L)	5.00 [4.10; 6.20]	5.00 [4.10; 6.20]	5.10 [4.00; 6.30]	0.214
CREA (umol/L)	62.00 [53.00; 72.00]	62.00 [53.00; 72.00]	62.00 [53.00; 72.00]	0.621
UA (umol/L)	262.00 [214.00; 314.00]	261.00 [214.00; 315.00]	262.00 [213.00; 311.00]	0.668
CYFRA211 (ng/mL)	4.81 [3.06; 9.49]	4.61 [2.98; 8.80]	6.47 [3.63; 14.96]	<0.001
NSE (ng/mL)	17.20 [13.31; 25.54]	16.77 [13.15; 24.13]	19.82 [14.29; 34.22]	<0.001
CEA (ng/mL)	5.00 [2.46; 22.48]	5.04 [2.48; 22.91]	4.80 [2.41; 20.47]	0.221
PT(s)	11.80 [11.20; 12.60]	11.80 [11.10; 12.50]	12.20 [11.60; 13.00]	<0.001
INR	0.97 [0.92; 1.03]	0.97 [0.92; 1.03]	1.01 [0.95; 1.07]	<0.001
PT_1 (%)	97.30 [90.20; 104.10]	97.30 [90.80; 104.70]	94.10 [86.70; 101.70]	<0.001
APTT(s)	29.00 [25.90; 32.90]	28.80 [25.80; 32.60]	29.70 [26.30; 33.90]	<0.001
TT(s)	16.60 [15.70; 17.50]	16.60 [15.70; 17.50]	16.40 [15.40; 17.40]	<0.001
FIB (g/L)	3.35 [2.68; 4.18]	3.32 [2.66; 4.13]	3.59 [2.79; 4.39]	<0.001
D-Dimmer (mg/L)	0.68 [0.40; 1.30]	0.61 [0.38; 1.12]	1.00 [0.49; 3.00]	<0.001

NSCLC, non-small cell lung cancer; SCLC, small cell lung cancer; HF, heart failure; COPD, chronic obstructive pulmonary disease; IPC, intermittent pneumatic compression; CVC, central venous catheter; WBC, white blood cell; RBC, red blood cell; Hb, Hemoglobin; PLT, Platelet Count; NEUT%, neutrophil percentage; LYM%, lymphocyte percentage; MONO%, monocyte percentage; ESO%, eosinophil percentage; BASO%, basophil percentage; NEUT, neutrophil count; LYM, lymphocyte count; MONO, monocyte count; ESO, eosinophil count; BASO, basophil count; HCT, hematocrit; MCV, mean corpuscular volume; MCH, mean corpuscular hemoglobin; MCHC, mean corpuscular hemoglobin concentration; RDW-SD, red blood cell distribution width standard deviation; RDW-CV, red blood cell distribution width coefficient of variation; PDW, platelet distribution width; MPV, mean platelet volume; PCT, plateletcrit; LPR, large platelet ratio; TBIL, total bilirubin; DBIL, direct bilirubin; IBIL, indirect bilirubin; ALT, alanine aminotransferase; AST, aspartate aminotransferase; ALP, alkaline phosphatase; GTT, gamma-glutamyl transferase; TP, total protein; ALB, albumin; GLOB, globulins; A/G, albumin/globulin ratio; BUN, blood urea nitrogen; Cr, creatinine; UA, uric acid; CYFRA211, tumor marker CYFRA211; NSE, tumor marker NSE; CEA, carcinoembryonic antigen; PT, prothrombin time; INR, international normalized ratio; PT_1, PT activity; APTT, activated partial thromboplastin time; TT, thrombin time; FIB, fibrinogen.

### 3.2 Selected predictors and construction model

**Figure 2:**
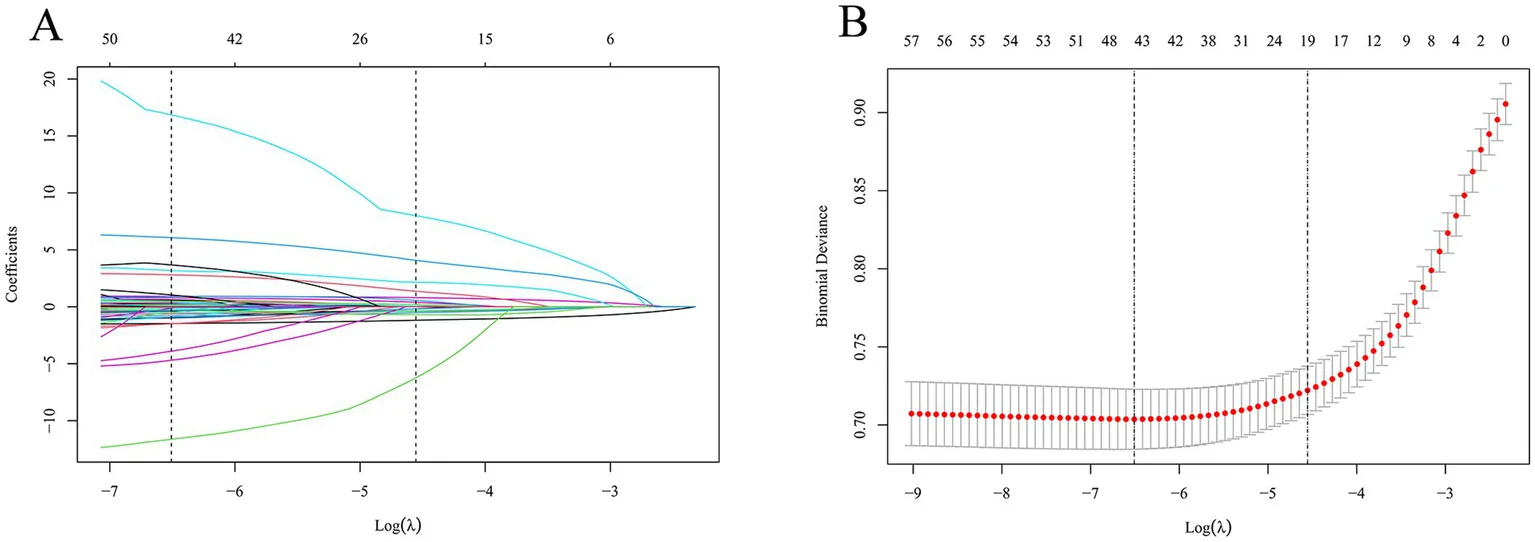
Feature selection using least absolute shrinkage and selection operator (LASSO) regression in the training cohort. **(A)** LASSO coefficient profiles of the candidate predictors. The x-axis represents the L1 norm, which reflects the degree of regularisation in the model. **(B)** Selection of the optimal tuning parameter (*λ*) in the LASSO model using 10-fold cross-validation. The dotted vertical lines indicate the minimum criterion and the one-standard-error criterion (λ.1se).

A total of 67 clinical variables were initially extracted from the EMRs as candidate predictors. In the training cohort, univariate analysis identified 22 variables associated with VTE (*p* < 0.05). Multivariable logistic regression retained 16 independent predictors (Supplementary Table 2), whereas LASSO regression with 10-fold cross-validation identified 19 variables with non-zero coefficients at *λ*.1se = 0.011 (Figures 2A,B). The overlap between the predictors identified by the two methods is shown in Supplementary Table 3. Twelve predictors were consistently selected by both methods and were therefore included in subsequent model development: sex, age, anticoagulant use, atherosclerosis, chemotherapy, IPC, radiotherapy, CYFRA21-1, neuron-specific enolase (NSE), CVC placement, thrombin time (TT), and D-dimer.

### 3.3 Model development and comparative performance

**Figure 3:**
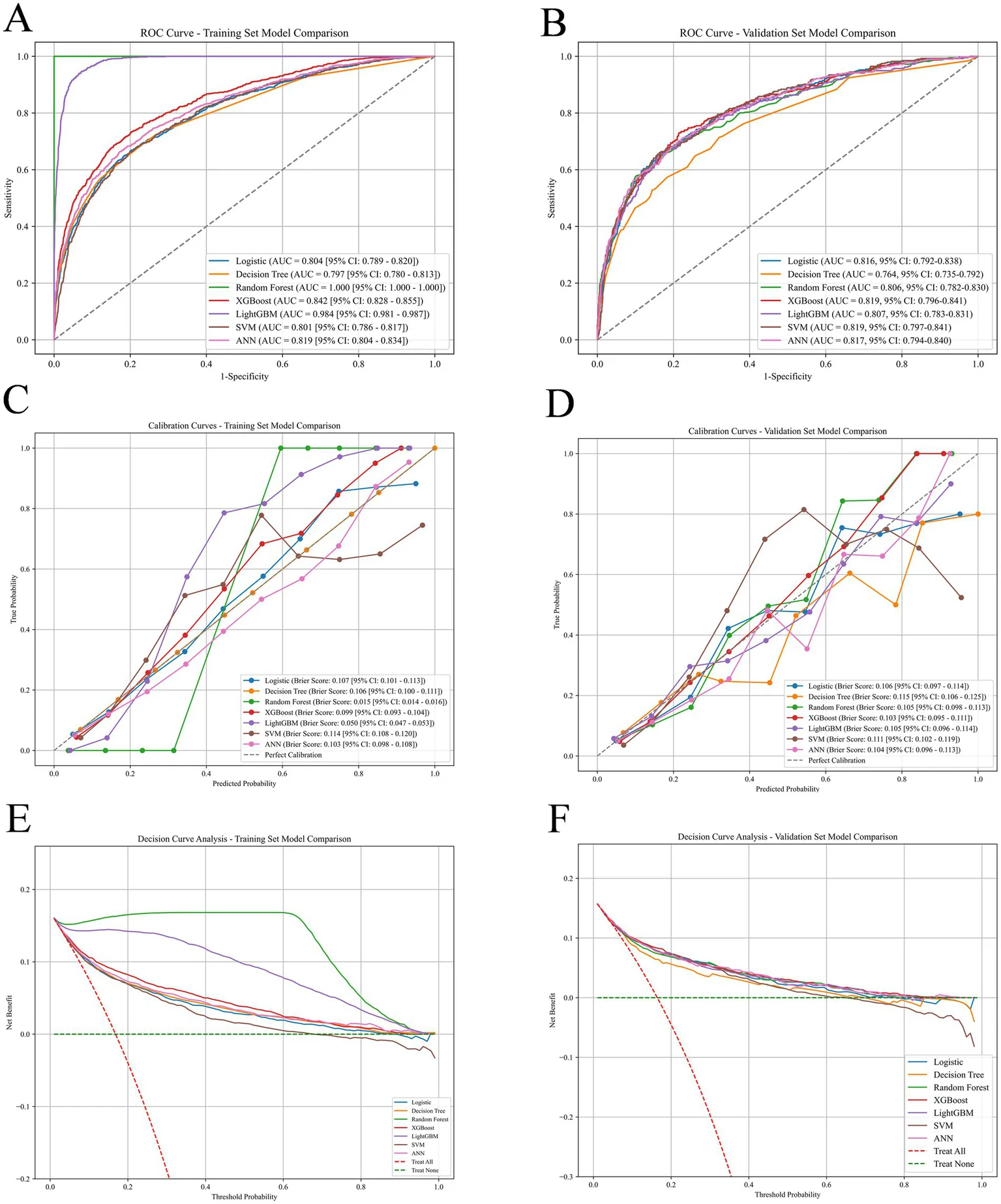
Performance of the seven prediction models in the training and testing cohorts. Receiver operating characteristic (ROC) curves of the seven models in the training cohort **(A)** and testing cohort **(B)**. Calibration curves of the seven models in the training cohort **(C)** and testing cohort **(D)**. Decision curve analysis (DCA) curves of the seven models in the training cohort **(E)** and testing cohort **(F)**. The seven models included logistic regression (LR), Decision Tree (DT), Random Forest (RF), Extreme Gradient Boosting (XGBoost), Light Gradient Boosting Machine (LightGBM), Support Vector Machine (SVM), and Artificial Neural Network (ANN).

Based on the 12 selected predictors, seven prediction models were developed in the training cohort, including six ML models and one LR model. In the training cohort, five-fold cross-validation based on receiver operating characteristic (ROC) analysis was performed to assess model discrimination and consistency across folds, with the results presented as mean AUROC ± SD in Supplementary Figure 1. The ROC curves, calibration curves, and decision curve analyses for all seven s in the training and testing cohorts are shown in Figures 3A–F. Detailed performance metrics of the seven models in the testing cohort are summarized in Table 3.

**Table 3:** Model evaluation metrics.

Model	AUROC	Accuracy	Precision	Sensitivity	Specificity	F1 Score	Kappa	Youden’s J	PPV	NPV
LR	0.816	0.854	0.586	0.404	0.943	0.478	0.397	0.347	0.586	0.888
DT	0.763	0.842	0.533	0.389	0.932	0.450	0.360	0.321	0.533	0.885
RF	0.806	0.860	0.620	0.404	0.951	0.489	0.412	0.355	0.620	0.889
XGBoost	0.819	0.861	0.625	0.412	0.951	0.496	0.420	0.362	0.625	0.890
LightGBM	0.807	0.854	0.585	0.419	0.941	0.488	0.406	0.360	0.585	0.891
SVM	0.819	0.860	0.704	0.270	0.977	0.391	0.329	0.248	0.704	0.871
ANN	0.817	0.856	0.576	0.505	0.926	0.538	0.454	0.431	0.576	0.904

LR, logistic regression; DT, Decision Tree; RF, Random Forest; XGBoost, Extreme Gradient Boosting; LightGBM, Light Gradient Boosting machine; SVM, Support Vector Machine; ANN, Artificial Neural Network; AUROC, the area under the Receiver Operating Characteristic ROC curve; PPV, positive predictive value; NPV, negative predictive value.

In the testing cohort, the AUROC (95% CI) was 0.816 (0.792–0.838) for LR, 0.764 (0.735–0.792) for DT, 0.806 (0.782–0.830) for RF, 0.819 (0.796–0.841) for XGBoost, 0.807 (0.783–0.831) for LightGBM, 0.819 (0.797–0.841) for SVM, and 0.817 (0.794–0.840) for ANN. The corresponding F1 scores were 0.478, 0.450, 0.489, 0.496, 0.488, 0.391, and 0.538, respectively.

Although XGBoost, SVM, and ANN showed similar AUROC values, ANN was selected as the final model because it achieved the highest F1 score in the testing cohort, indicating the most favorable balance between sensitivity and precision for identifying patients at high risk of VTE. ANN also showed the highest mean cross-validated AUROC in the training cohort (0.825 ± 0.016), further supporting its robustness and consistency across folds.

### 3.4 Calibration and clinical utility

Calibration curves for the seven models are shown in Figures 3C,D. Overall, the predicted probabilities were generally consistent with the observed outcomes. In the testing cohort, the Brier scores were 0.106 for LR, 0.115 for DT, 0.105 for RF, 0.103 for XGBoost, 0.105 for LightGBM, 0.111 for SVM, and 0.104 for ANN. These results suggested comparable calibration across the higher-performing models, with ANN showing calibration similar to that of XGBoost, RF, and LightGBM.

The DCA curves are shown in Figures 3E,F. In both the training and testing cohorts, all models showed positive net benefit over threshold probabilities of approximately 0.0–0.40. Across this range, ANN showed higher net benefit than LR, DT, RF, and SVM, and broadly similar net benefit to XGBoost and LightGBM, supporting its potential clinical utility in identifying patients at high risk of VTE.

**Figure 4:**
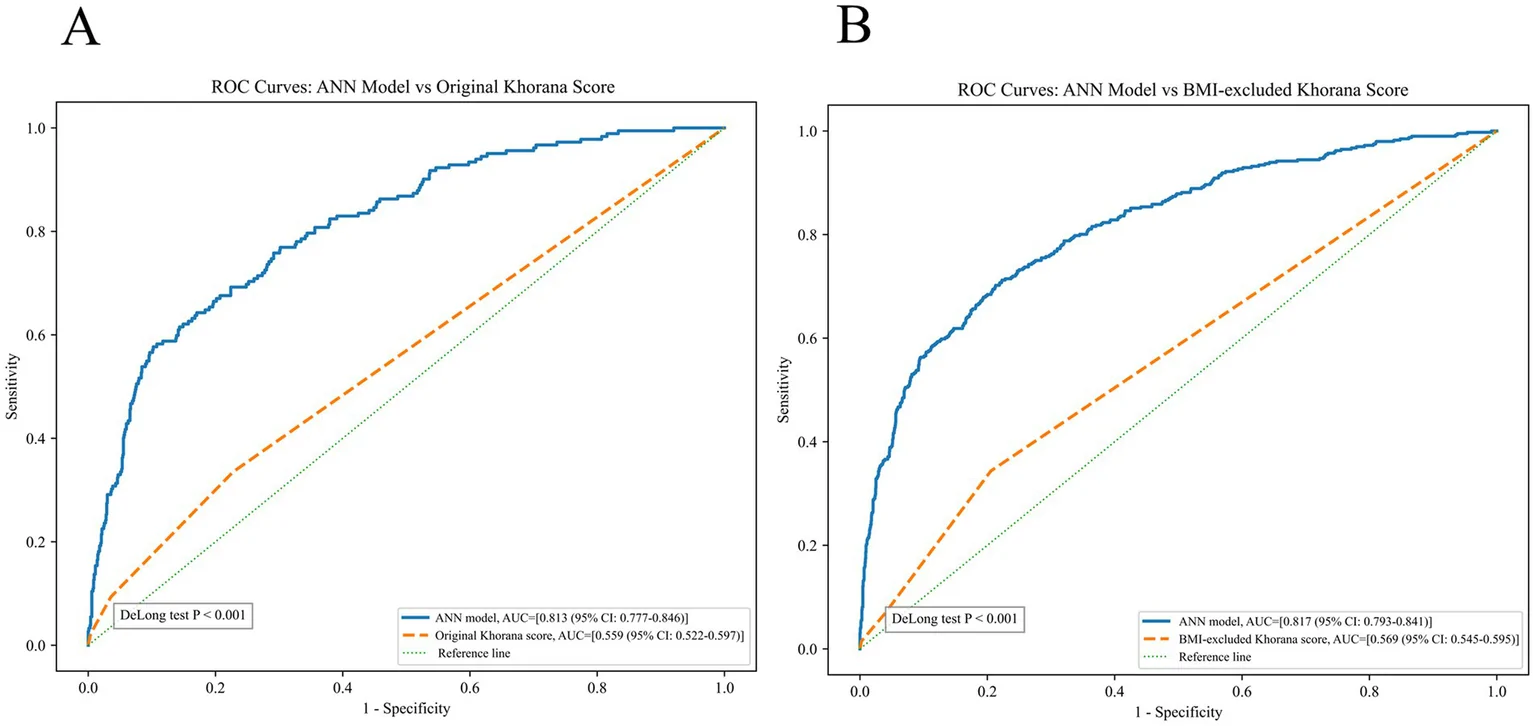
Receiver operating characteristic curves comparing the ANN model with Khorana-based scores. **(A)** Comparison between the ANN model and the original Khorana score in the BMI-complete testing subset. **(B)** Sensitivity analysis comparing the ANN model with the BMI-excluded Khorana score in the testing cohort. The BMI-excluded Khorana score was calculated by summing all original Khorana components except the BMI criterion and was used only as an exploratory sensitivity analysis. AUCs with 95% confidence intervals are shown in each panel. Differences between AUCs were assessed using the DeLong test.

To further assess the added value of the ANN model, we compared its discriminative performance with that of the Khorana score in the validation cohort. In the primary analysis restricted to the BMI-complete validation subset, 1249 patients were included, among whom 182 developed VTE. The ANN model achieved an AUC of 0.813 (95% CI: 0.777–0.846), which was significantly higher than that of the original Khorana score (AUC: 0.559; 95% CI: 0.552–0.597; DeLong test, *p* < 0.001; Figure 4A).

Given the high missing rate of BMI, we further performed a sensitivity analysis using the BMI-excluded Khorana score. This analysis included 2387 patients, among whom 396 developed VTE. The ANN model maintained good discriminative performance, with an AUC of 0.817 (95% CI: 0.793–0.841), whereas the BMI-excluded Khorana score showed limited discrimination, with an AUC of 0.569 (95% CI: 0.545–0.595). The difference in AUC remained statistically significant (DeLong test, *p* < 0.001; Figure 4B). These findings indicate that the ANN model showed better discriminative performance than the Khorana-based scores in both the primary and sensitivity analyses.

### 3.5 Model interpretation

SHAP analysis was performed on the final ANN model to provide both global and individual-level interpretation of model predictions.

**Figure 5:**
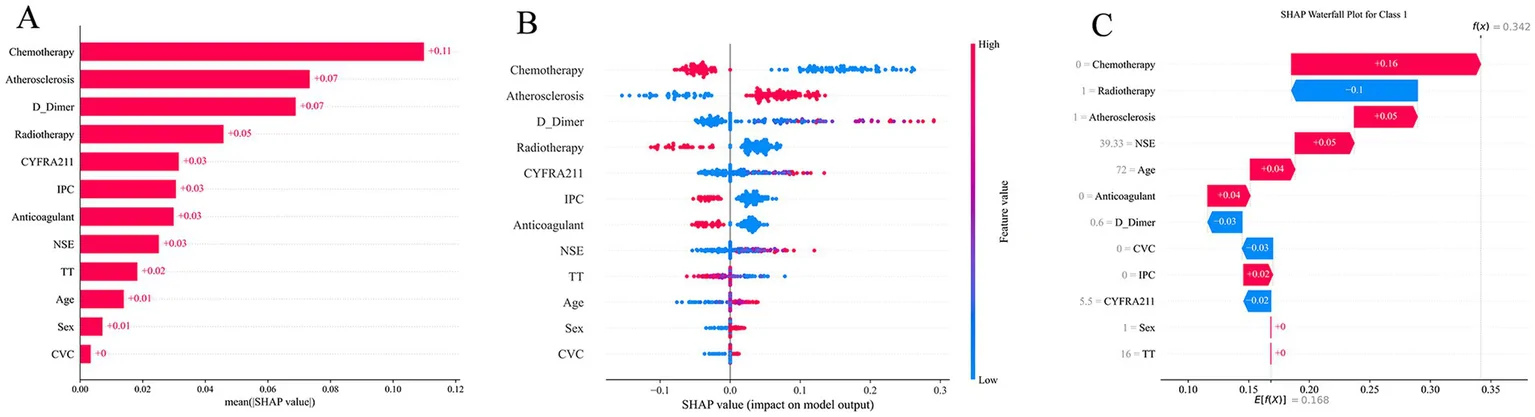
SHAP-based interpretation of the final ANN model VTE risk prediction. **(A)** Bar plot of the mean absolute SHAP values, indicating the relative importance of each predictor. **(B)** SHAP beeswarm plot showing the distribution and direction of the effects of the predictors on model output. **(C)** Local SHAP explanation for a representative patient with high predicted VTE risk.

At the global level, the SHAP summary bar plot ranked the 12 predictors according to their mean absolute SHAP values (Figure 5A). The five most influential variables were chemotherapy, atherosclerosis, D-dimer, radiotherapy, and CYFRA21-1. The SHAP summary beeswarm plot illustrated the distribution, direction, and magnitude of SHAP values for each predictor (Figure 5B). Higher values of D-dimer, CYFRA21-1, and NSE tended to correspond to positive SHAP values, whereas lower values corresponded to negative SHAP values. For binary predictors such as atherosclerosis and IPC, the presence of the feature was more frequently linked to positive SHAP values. SHAP dependence plots for all 12 predictors were presented in Supplementary Figure 2, showing the relationship between feature values and their corresponding contributions across the study population.

At the individual level, SHAP waterfall plots illustrated how specific predictors contributed to each patient’s predicted risk. For patient #3 (Figure 5C), atherosclerosis and age (72 years) were associated with positive SHAP values of +0.05 and +0.04, respectively, whereas D-dimer (0.6 mg/L) was associated with a negative SHAP value of −0.03. Other predictors, including NSE, CVC, CYFRA21-1, and IPC, contributed cumulatively to the final predicted risk. The waterfall plot effectively visualized the combined influence of these variables for individual-level prediction.

## 4 Discussion

We developed and validated a ML–based model to predict VTE risk in patients with lung cancer. Seven algorithms were evaluated, and the final model incorporated 12 clinically accessible predictors: sex, age, anticoagulant use, atherosclerosis, chemotherapy, IPC, radiotherapy, CYFRA21-1, NSE, CVC placement, TT, and D-dimer. All predictors can be readily extracted from EMRs or routine laboratory tests, facilitating potential integration into clinical workflows. To enhance interpretability, SHAP was applied to visualize each variable’s contribution to individual predictions.

In model comparisons, XGBoost achieved a slightly higher AUROC in the testing cohort, whereas ANN yielded the highest F1 score, indicating a more favorable balance between sensitivity and precision. ANN also showed the highest mean cross-validated AUROC in the training cohort, supporting its robustness and consistency. In contrast, some highly flexible models, such as RF, showed extremely high apparent performance in the training cohort but lower performance in the testing cohort, suggesting potential overfitting. Therefore, the final model was not selected solely according to training performance, but was determined by considering testing performance, cross-validation results, and the consistency between training and testing performance. This may reflect the ability of ANN to capture complex nonlinear relationships among predictors while showing a more balanced performance profile than models with marked training–testing discrepancies. Given the clinical importance of identifying patients at higher predicted risk, ANN may offer advantages for risk stratification, although further external validation is required.

The *post hoc* comparison with the Khorana score further supported the added value of the ANN model. The ANN model outperformed the original Khorana score in the BMI-complete validation subset, and this finding remained consistent in the sensitivity analysis using the BMI-excluded Khorana score. These results suggest that the ANN model may better capture the multifactorial and nonlinear nature of VTE risk in patients with cancer than a simple additive risk score. However, the BMI-excluded Khorana score was used only as an exploratory sensitivity analysis and should not be regarded as a validated alternative to the original Khorana score. Therefore, these findings should be interpreted as evidence of improved discrimination within this cohort, and further external validation is needed.

Several VTE risk scores are currently available, such as the Caprini score for surgical patients (18) and the Padua prediction score for medical patients (19). Although the Khorana score was specifically developed for cancer patients in 2008 and has been widely used (9), its discriminative ability remains limited (20), particularly in lung cancer (11). By integrating additional clinically relevant variables and nonlinear modeling, our model may better reflect the heterogeneous and multidimensional nature of VTE risk in this population, and therefore may provide additional predictive value beyond traditional scoring systems.

Interpretability remains a major challenge in the clinical application of ML. In previous ML-based studies on VTE, only a limited proportion used SHAP for model interpretation (12). Recent oncology studies have increasingly incorporated SHAP into ML-based prediction models to improve model transparency and clinical interpretability. For example, SHAP-based models have been used to interpret predictors of long-term recurrence after breast-conserving surgery for ductal carcinoma *in situ* and to explain treatment response and long-term outcomes in patients with breast cancer receiving neoadjuvant chemotherapy (21, 22). In the present study, SHAP was applied to assess global feature importance and to provide local explanations at the individual level. In particular, SHAP dependence plots enabled visualization of nonlinear patterns and potential threshold effects that might not be captured by conventional linear or univariable approaches (23). For example, SHAP dependence plots provided additional information on the nonlinear relationship between D-dimer and predicted VTE risk. These findings support the value of SHAP as an interpretability framework for translating complex ML models into clinically understandable risk profiles. Nevertheless, SHAP-based explanations should be interpreted as model-level explanations rather than evidence of causal relationships.

Previous studies have shown inconsistent findings regarding the association between D-dimer and VTE risk. Some studies found that preoperative D-dimer elevation was not predictive of postoperative deep venous thrombosis (24), whereas others suggested that perioperative elevation may be associated with VTE occurrence (25) or recurrence (26). More recent studies have highlighted the importance of D-dimer dynamics (27). In our study, SHAP dependence plots suggested a nonlinear association between D-dimer and predicted VTE risk. Predicted risk generally increased when D-dimer exceeded approximately 2 mg/L, but the rate of increase gradually attenuated at higher levels and appeared to plateau at around 23 mg/L. These findings suggest that D-dimer may provide more nuanced risk information than a simple dichotomous interpretation, although the specific threshold values and their clinical applicability require further validation.

Anticoagulant use was associated with lower predicted VTE risk in our model. However, this finding should be interpreted cautiously because anticoagulant use represents a treatment-related exposure rather than a simple baseline characteristic. In this study, anticoagulant use was defined as pre-VTE anticoagulant-related exposure documented in the medical records and should not be interpreted as a strictly defined pharmacologic VTE chemoprophylaxis variable. In routine practice, prophylactic anticoagulation may be prescribed to patients perceived to be at higher baseline risk (28), and it may also be modified or withheld according to bleeding risk, procedures, platelet counts, or other clinical considerations. Therefore, this variable may reflect both clinician-guided treatment selection and treatment effects, leading to possible confounding by indication. Unlike previous studies that excluded anticoagulated patients or included only anticoagulated patients, our cohort included both anticoagulated and non-anticoagulated individuals, allowing the model to capture the more complex role of anticoagulant use in real-world clinical practice. Nevertheless, this association should not be interpreted as evidence of a direct causal effect. In addition, because of the retrospective design, we could not consistently distinguish prescribed-but-held anticoagulation, actual inpatient pharmacologic prophylaxis, chronic anticoagulant therapy, or discharge prophylaxis. The lack of detailed information on anticoagulant type, dose, timing and duration limited further interpretation. Future prospective studies should collect anticoagulation-related variables in more granular categories to better clarify their relationship with VTE risk.

In our cohort, different cancer treatments and invasive procedures were associated with variations in predicted VTE risk. Surgery was not identified as a significant predictor, whereas chemotherapy and radiotherapy were associated with lower predicted risk. This finding should be interpreted with caution, particularly for chemotherapy, because previous studies have generally reported an increased risk of VTE among patients receiving chemotherapy. One possible explanation for this discrepancy is that our dataset lacked information on tumor stage, targeted therapy, and immunotherapy. This limitation is important because patients who did not receive chemotherapy or other systemic treatments may have been more likely to have advanced-stage disease, and advanced-stage disease is generally associated with a higher risk of VTE (29). In parallel, both targeted therapy and immunotherapy have also been reported to be associated with increased VTE risk in patients with lung cancer (30, 31). Therefore, the observed associations of chemotherapy and radiotherapy with lower predicted risk may reflect differences in baseline risk related to disease stage, treatment selection, and other unmeasured confounders, rather than the direct effects of these treatments themselves.

CVC placement is a recognized risk factor for thrombosis (32). A survey of healthcare professionals reported that most respondents (76.2%) considered CVC placement to be associated with an increased risk of thrombosis (33). Consistently, in our study, CVC placement was associated with higher predicted VTE risk. By contrast, IPC was associated with lower predicted VTE risk. This is in line with previous evidence supporting its role in thromboprophylaxis (34).

Previous studies have reported an association between atherosclerosis and venous thrombosis, including links between asymptomatic carotid atherosclerotic plaques and spontaneous lower-limb VTE (35), coronary artery disease and VTE risk (36), and myocardial infarction and a transient increase in VTE risk (37). A possible explanation is that atherosclerosis and VTE share common risk factors, such as older age, obesity, diabetes mellitus, and metabolic syndrome, and may also involve overlapping pathogenic mechanisms (38, 39). However, the evidence remains inconsistent. Some studies have shown that carotid atherosclerosis is strongly associated with future myocardial infarction but not with VTE (40). In our cohort, atherosclerosis was associated with higher predicted VTE risk, whereas prior myocardial infarction and ischemic stroke were not. This finding suggests that the increased VTE risk observed in our lung cancer cohort may be more closely related to chronic atherosclerotic burden than to acute arterial events.

Age and sex were associated with predicted VTE risk in our model. Previous studies have suggested that the effect of sex may vary across age groups, with higher risk observed in younger women (<50 years) and older men (≥65 years) (41). In our cohort, which consisted predominantly of older male patients (63.0% male, 30.9% aged ≥65 years), male sex and increasing age were associated with higher predicted VTE risk. This pattern may partly reflect the higher prevalence of smoking and comorbidities in these subgroups (39, 42, 43).

CYFRA 21-1 and NSE were also positively associated with predicted VTE risk. Direct evidence linking these tumor markers to VTE remains limited. Their elevation may reflect greater tumor burden or more aggressive tumor biology, both of which may contribute to systemic activation of coagulation (29, 44–50). These findings suggest that CYFRA 21-1 and NSE may provide additional information for identifying patients at higher predicted risk of VTE and may warrant closer clinical attention.

TNM staging was excluded from model development because of its high missing rate, and this should be considered when interpreting the model. TNM stage captures important aspects of disease severity, including primary tumor extent, nodal involvement, and distant metastasis. Prior studies have shown that patients with regional lymph node involvement or distant metastatic disease have a higher risk of VTE than those with localized disease, and this association may be partly related to tumor-driven activation of coagulation and fibrinolytic pathways, systemic inflammatory responses, and treatment-related factors (51, 52). Therefore, the exclusion of TNM staging may have limited the model’s ability to fully capture disease burden and may have introduced residual confounding. Moreover, the estimated contributions of correlated variables, such as chemotherapy, CVC placement, laboratory abnormalities, or other treatment-related factors, may have been influenced because these variables could partly act as proxies for advanced disease. Future studies with more complete TNM staging information are needed to determine whether incorporating TNM stage can improve model discrimination, calibration, and clinical interpretability.

In addition to missing TNM staging, the timing and clinical meaning of treatment-related predictors should also be considered when interpreting the model. The model was not designed as a purely baseline admission risk model, because several predictors, including anticoagulant use, chemotherapy, radiotherapy, IPC, and CVC placement, were treatment- or procedure-related exposures documented before VTE occurrence. Although post-VTE information was excluded, these variables may partly reflect clinical decision-making, disease severity, or perceived thrombotic risk, and residual temporal bias or confounding by indication cannot be completely ruled out. Prospective studies with prespecified predictor assessment windows are needed to further validate the model and clarify its potential role as a dynamic clinical prediction tool.

This study has several limitations. First, its retrospective observational design makes it vulnerable to uncontrolled confounding and precludes causal inference. In addition, this was a single-center retrospective study, and the model was developed and internally validated using a random training/testing split from the same institution. Although internal validation provides preliminary evidence of model performance, it does not fully establish generalizability across different institutions, patient populations, clinical workflows, treatment patterns, laboratory measurements, or VTE diagnostic practices. Therefore, external validation in independent cohorts, preferably prospective multicenter cohorts is required before broader clinical implementation. Second, several data constraints may have affected model development and interpretion, including the exclusion of TNM staging because of substantial missingness, the absence of detailed anticoagulation data, and incomplete information on targeted therapy and immunotherapy. Third, although treatment- and procedure-related predictors were defined as pre-event exposures and post-VTE information was excluded, these variables may still partly reflect clinical decision-making, disease severity, or perceived thrombotic risk. Therefore, residual temporal bias and confounding by indication cannot be completely excluded. Fourth, although predictor selection was performed using multivariable logistic regression and LASSO regression, and model tuning was conducted with cross-validation, residual overfitting cannot be fully excluded. Finally, cancer-related death within 180 days may have acted as a competing event for VTE, but death was not explicitly modeled as a competing risk in the current binary prediction framework. Future prospective multicenter studies with more comprehensive data and time-to-event outcomes are needed to further assess predictive performance, confirm model stability, and refine clinical utility.

Overall, our findings suggest that VTE risk assessment in patients with lung cancer may be improved by integrating demographic, clinical, and laboratory variables within a ML framework. The final model, based on 12 routinely available predictors, offers an interpretable approach for individualized risk estimation and may offer additional value beyond conventional scoring systems. In *post hoc* benchmark analyses, the ANN model showed better discriminative performance than Khorana-based scores, further supporting its potential value for VTE risk stratification. However, further external validation, preferably in prospective multicenter cohorts, is needed before implementation in broader clinical practice.

## Data Availability

The raw data supporting the conclusions of this article will be made available by the authors, without undue reservation.
