# Correction: Development and validation of an interpretable machine learning model for venous thromboembolism risk prediction in patients with lung cancer: a real-world study

**DOI:** 10.3389/fmed.2026.1953862

**Published:** 2026-09-08

**Authors:** Frontiers Production Office

**Affiliations:** Frontiers Media SA, Lausanne, Switzerland

**Keywords:** lung cancer, machine learning, risk prediction, SHapley Additive exPlanation, venous thromboembolism

There was a mistake in **Table 1** as published. The final *P-*values for both “Anticoagulant” and “Chemotherapy drugs” in **Table 1** have been corrected to 1.000.

**Table 1** presents the comparison of baseline characteristics between the training and validation cohorts in the clinical prediction model. These *P-*values were used to assess whether there were statistically significant differences between the two cohorts for the relevant baseline variables. Although the published and corrected *P*-values are different, both are greater than 0.05. Therefore, the statistical interpretation remains unchanged, indicating that no statistically significant differences were observed between the training and validation cohorts for these two variables.

This correction is limited to the accuracy of the *P*-values reported in **Table 1** and does not affect the model construction, results, interpretation, or conclusions of the article.

The original version of this article has been updated.

