# Supplementary material for: Development and validation of an interpretable machine learning model for venous thromboembolism risk prediction in patients with lung cancer: a real-world study
Source: Front Med (Lausanne). 2026 Jul 8;13:1853920. doi: 10.3389/fmed.2026.1853920 (PMC13388784; doi:10.3389/fmed.2026.1853920)
Supplement: Supplementary file 1 [file Table_1.DOCX]

Supplementary table 1. Percentage of missing data in each variable.

| Variable | Missing rate (%) |
| --- | --- |
| Sex | 0 |
| BMI | 49 |
| ECOG | 50 |
| TNM stage | 54 |
| Operation | 0 |
| Pathology | 4 |
| Hypertension | 0 |
| Diabetes | 0 |
| Cerebral infarction | 0 |
| Cerebral hemorrhage | 0 |
| Hemostatic drugs | 0 |
| Coronary heart disease | 0 |
| HF | 0 |
| COPD | 0 |
| Anticoagulants | 0 |
| Atherosclerosis | 0 |
| Chemotherapy drugs | 0 |
| IPC | 0 |
| Vasopressors | 0 |
| Radiotherapy | 0 |
| CVC | 0 |
| Age | 0 |
| WBC | 2 |
| RBC | 2 |
| Hb | 2 |
| PLT | 2 |
| NEUT_1 | 2 |
| LYMPH_1 | 2 |
| MONO_1 | 2 |
| EOS_1 | 2 |
| BASO_1 | 2 |
| NEUT | 2 |
| LYMPH | 2 |
| MONO | 2 |
| EOS | 2 |
| BASO | 2 |
| HCT | 2 |
| MCV | 2 |
| MCH | 2 |
| MCHC | 2 |
| RDW_SD | 2 |
| RDW_CV | 2 |
| PDW | 3 |
| MPV | 3 |
| PCT | 3 |
| P_LCR | 3 |
| CRP | 63 |
| TBIL | 2 |
| DBIL | 2 |
| IBIL | 2 |
| ALT | 2 |
| AST | 2 |
| ALP | 2 |
| GGT | 2 |
| TP | 2 |
| ALB | 2 |
| GLOB | 2 |
| A_G | 2 |
| BUN | 2 |
| CREA | 2 |
| UA | 8 |
| CYFRA211 | 13 |
| NSE | 12 |
| CEA | 11 |
| PT | 3 |
| INR | 3 |
| PT_1 | 3 |
| APTT | 3 |
| TT | 3 |
| FBG | 3 |
| D-Dimer | 20 |

Abbreviations: NSCLC: non-small cell lung cancer ,SCLC: small cell lung cancer, HF: heart failure, COPD: chronic obstructive pulmonary disease, IPC: intermittent pneumatic compression, CVC: central venous catheter, WBC: white blood cell, RBC: red blood cell, Hb: Hemoglobin, PLT: Platelet Count, NEUT%: neutrophil percentage, LYM%: lymphocyte percentage, MONO%: monocyte percentage, ESO%: eosinophil percentage, BASO%: basophil percentage, NEUT: neutrophil count , LYM: lymphocyte count , MONO: monocyte count, ESO: eosinophil count, BASO: basophil count, HCT: hematocrit, MCV: mean corpuscular volume, MCH: mean corpuscular hemoglobin, MCHC: mean corpuscular hemoglobin concentration, RDW-SD: red blood cell distribution width standard deviation, RDW-CV: red blood cell distribution width coefficient of variation, PDW: platelet distribution width, MPV: mean platelet volume, PCT: plateletcrit, LPR: large platelet ratio, TBIL: total bilirubin, DBIL: direct bilirubin, IBIL: indirect bilirubin, ALT: alanine aminotransferase, AST: aspartate aminotransferase, ALP: alkaline phosphatase, GTT: gamma-glutamyl transferase, TP: total protein, ALB: albumin, GLOB: globulins, A/G: albumin/globulin ratio, BUN: blood urea nitrogen, Cr: creatinine, UA: uric acid, CYFRA211: tumor marker CYFRA211, NSE: tumor marker NSE, CEA: carcinoembryonic antigen, PT: prothrombin time, INR: international normalized ratio, PT_1: PT activity, APTT: activated partial thromboplastin time, TT: thrombin time, FIB：fibrinogen.
