# Supplementary material for: Development and validation of an interpretable machine learning model for venous thromboembolism risk prediction in patients with lung cancer: a real-world study
Source: Front Med (Lausanne). 2026 Jul 8;13:1853920. doi: 10.3389/fmed.2026.1853920 (PMC13388784; doi:10.3389/fmed.2026.1853920)
Supplement: Supplementary file 2 [file Table_2.DOCX]

Supplementary Table 2. Multivariable logistic regression analysis of risk factors for VTE in patients with lung cancer.

| Variable | β | SE | OR | CI | *P* value |
| --- | --- | --- | --- | --- | --- |
| Sex (male vs female) | 0.265 | 0.107 | 1.304 | 1.056-1.609 | 0.014 |
| Anticoagulant (yes vs no) | -0.528 | 0.092 | 0.59 | 0.492-0.707 | ＜0.001 |
| Atherosclerosis (yes vs no) | 0.958 | 0.091 | 2.606 | 2.179-3.118 | ＜0.001 |
| Chemotherapy drugs  (yes vs no) | -1.557 | 0.1 | 0.211 | 0.173-0.256 | ＜0.001 |
| IPC (yes vs no) | -0.678 | 0.099 | 0.507 | 0.418-0.616 | ＜0.001 |
| Radiotherapy (yes vs no) | -0.826 | 0.101 | 0.438 | 0.359-0.533 | ＜0.001 |
| CVC placement (yes vs no) | 0.555 | 0.108 | 1.741 | 1.409-2.151 | ＜0.001 |
| Age | 0.012 | 0.004 | 1.012 | 1.004-1.021 | 0.006 |
| PCT | -1.57 | 0.649 | 0.208 | 0.058-0.743 | 0.016 |
| ALP | -0.001 | 0 | 0.999 | 0.998-1.000 | 0.004 |
| CYFRA211 | 0.005 | 0.002 | 1.005 | 1.002-1.009 | 0.002 |
| NSE | 0.598 | 0.107 | 1.818 | 1.475-2.242 | ＜0.001 |
| PT_1 | 0.016 | 0.006 | 1.016 | 1.004-1.028 | 0.007 |
| APTT | -0.021 | 0.009 | 0.979 | 0.962-0.996 | 0.016 |
| TT | -0.175 | 0.032 | 0.84 | 0.788-0.895 | ＜0.001 |
| D_Dimer | 0.069 | 0.011 | 1.072 | 1.049-1.095 | ＜0.001 |

Abbreviations: IPC: intermittent pneumatic compression, CVC: central venous catheter, PCT: plateletcrit, ALP: alkaline phosphatase, CYFRA211: tumor marker CYFRA211, NSE: tumor marker NSE, PT_1: PT activity, APTT: activated partial thromboplastin time, TT: thrombin time.
