# Supplementary material for: Development and validation of an interpretable machine learning model for venous thromboembolism risk prediction in patients with lung cancer: a real-world study
Source: Front Med (Lausanne). 2026 Jul 8;13:1853920. doi: 10.3389/fmed.2026.1853920 (PMC13388784; doi:10.3389/fmed.2026.1853920)
Supplement: Supplementary file 3 [file Table_3.DOCX]

Supplementary Table 3. Predictors identified by multivariable logistic regression and LASSO regression and their overlap

| Variable | Multivariable logistic regression | LASSO regression | Selected by both methods |
| --- | --- | --- | --- |
| Sex | Yes | Yes | Yes |
| Operation |  |  |  |
| Pathology |  |  |  |
| Hypertension |  |  |  |
| Diabetes |  |  |  |
| Cerebral infarction |  |  |  |
| Cerebral hemorrhage |  |  |  |
| Hemostatic drugs |  |  |  |
| Coronary heart disease |  |  |  |
| HF |  |  |  |
| COPD |  |  |  |
| Anti coagulants | Yes | Yes | Yes |
| Atherosclerosis | Yes | Yes | Yes |
| Chemotherapy | Yes | Yes | Yes |
| IPC | Yes | Yes | Yes |
| Vasopressors |  |  |  |
| Radiotherapy | Yes | Yes | Yes |
| CVC placement | Yes | Yes | Yes |
| Age | Yes | Yes | Yes |
| WBC |  |  |  |
| RBC |  |  |  |
| Hb |  |  |  |
| PLT |  |  |  |
| NEUT_1 |  |  |  |
| LYMPH_1 |  | Yes |  |
| MONO_1 |  |  |  |
| EOS_1 |  |  |  |
| BASO_1 |  |  |  |
| NEUT |  | Yes |  |
| LYMPH |  |  |  |
| MONO |  |  |  |
| EOS |  |  |  |
| BASO |  |  |  |
| HCT |  |  |  |
| MCV |  |  |  |
| MCH |  |  |  |
| MCHC |  |  |  |
| RDW_SD |  | Yes |  |
| RDW_CV |  |  |  |
| PDW |  |  |  |
| MPV |  | Yes |  |
| PCT | Yes |  |  |
| P_LCR |  |  |  |
| TBIL |  |  |  |
| DBIL |  |  |  |
| IBIL |  |  |  |
| ALT |  |  |  |
| AST |  |  |  |
| ALP | Yes |  |  |
| GGT |  |  |  |
| TP |  |  |  |
| ALB |  | Yes |  |
| GLOB |  |  |  |
| A_G |  | Yes |  |
| BUN |  |  |  |
| CREA |  |  |  |
| UA |  |  |  |
| CYFRA211 | Yes | Yes | Yes |
| NSE | Yes | Yes | Yes |
| CEA |  |  |  |
| PT |  | Yes |  |
| INR |  |  |  |
| PT_1 | Yes |  |  |
| APTT | Yes |  |  |
| TT | Yes | Yes | Yes |
| FBG |  |  |  |
| D_Dimmer | Yes | Yes | Yes |

Abbreviations: NSCLC: non-small cell lung cancer ,SCLC: small cell lung cancer, HF: heart failure, COPD: chronic obstructive pulmonary disease, IPC: intermittent pneumatic compression, CVC: central venous catheter, WBC: white blood cell, RBC: red blood cell, Hb: Hemoglobin, PLT: Platelet Count, NEUT%: neutrophil percentage, LYM%: lymphocyte percentage, MONO%: monocyte percentage, ESO%: eosinophil percentage, BASO%: basophil percentage, NEUT: neutrophil count , LYM: lymphocyte count , MONO: monocyte count, ESO: eosinophil count, BASO: basophil count, HCT: hematocrit, MCV: mean corpuscular volume, MCH: mean corpuscular hemoglobin, MCHC: mean corpuscular hemoglobin concentration, RDW-SD: red blood cell distribution width standard deviation, RDW-CV: red blood cell distribution width coefficient of variation, PDW: platelet distribution width, MPV: mean platelet volume, PCT: plateletcrit, LPR: large platelet ratio, TBIL: total bilirubin, DBIL: direct bilirubin, IBIL: indirect bilirubin, ALT: alanine aminotransferase, AST: aspartate aminotransferase, ALP: alkaline phosphatase, GTT: gamma-glutamyl transferase, TP: total protein, ALB: albumin, GLOB: globulins, A/G: albumin/globulin ratio, BUN: blood urea nitrogen, Cr: creatinine, UA: uric acid, CYFRA211: tumor marker CYFRA211, NSE: tumor marker NSE, CEA: carcinoembryonic antigen, PT: prothrombin time, INR: international normalized ratio, PT_1: PT activity, APTT: activated partial thromboplastin time, TT: thrombin time, FIB：fibrinogen
