# Supplementary material for: Development and validation of an interpretable machine learning model for venous thromboembolism risk prediction in patients with lung cancer: a real-world study
Source: Front Med (Lausanne). 2026 Jul 8;13:1853920. doi: 10.3389/fmed.2026.1853920 (PMC13388784; doi:10.3389/fmed.2026.1853920)
Supplement: Supplementary file 4 [file Table_4.DOCX]

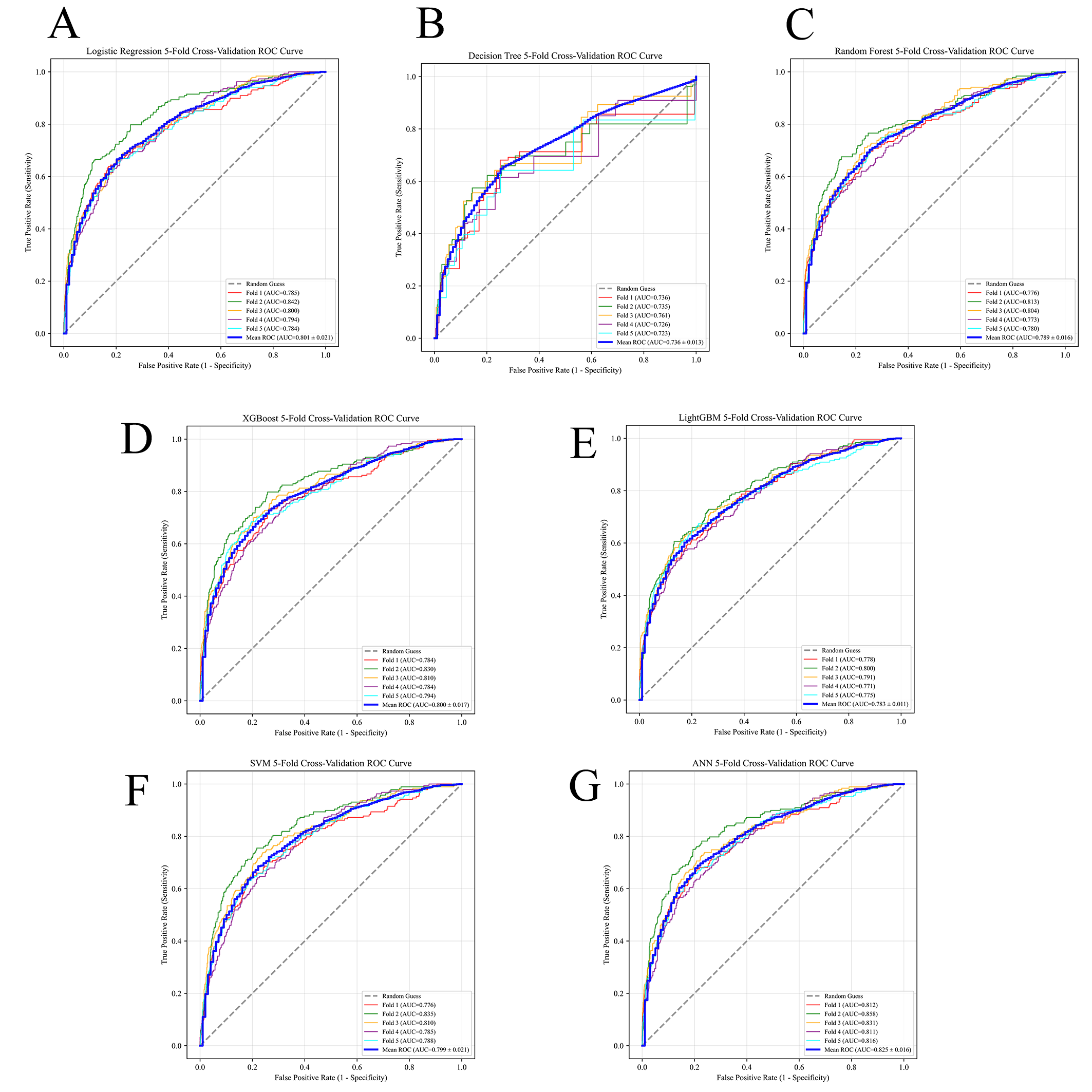


Supplementary Figure 1. Five-fold cross-validation receiver operating characteristic (ROC) curves for the seven prediction models in the training cohort. (A) logistic regression (LR); (B) Decision Tree (DT); (C) Random Forest (RF); (D) Extreme Gradient Boosting (XGBoost); (E) Light Gradient Boosting Machine (LightGBM); (F) Support Vector Machine (SVM); and (G) Artificial Neural Network (ANN).


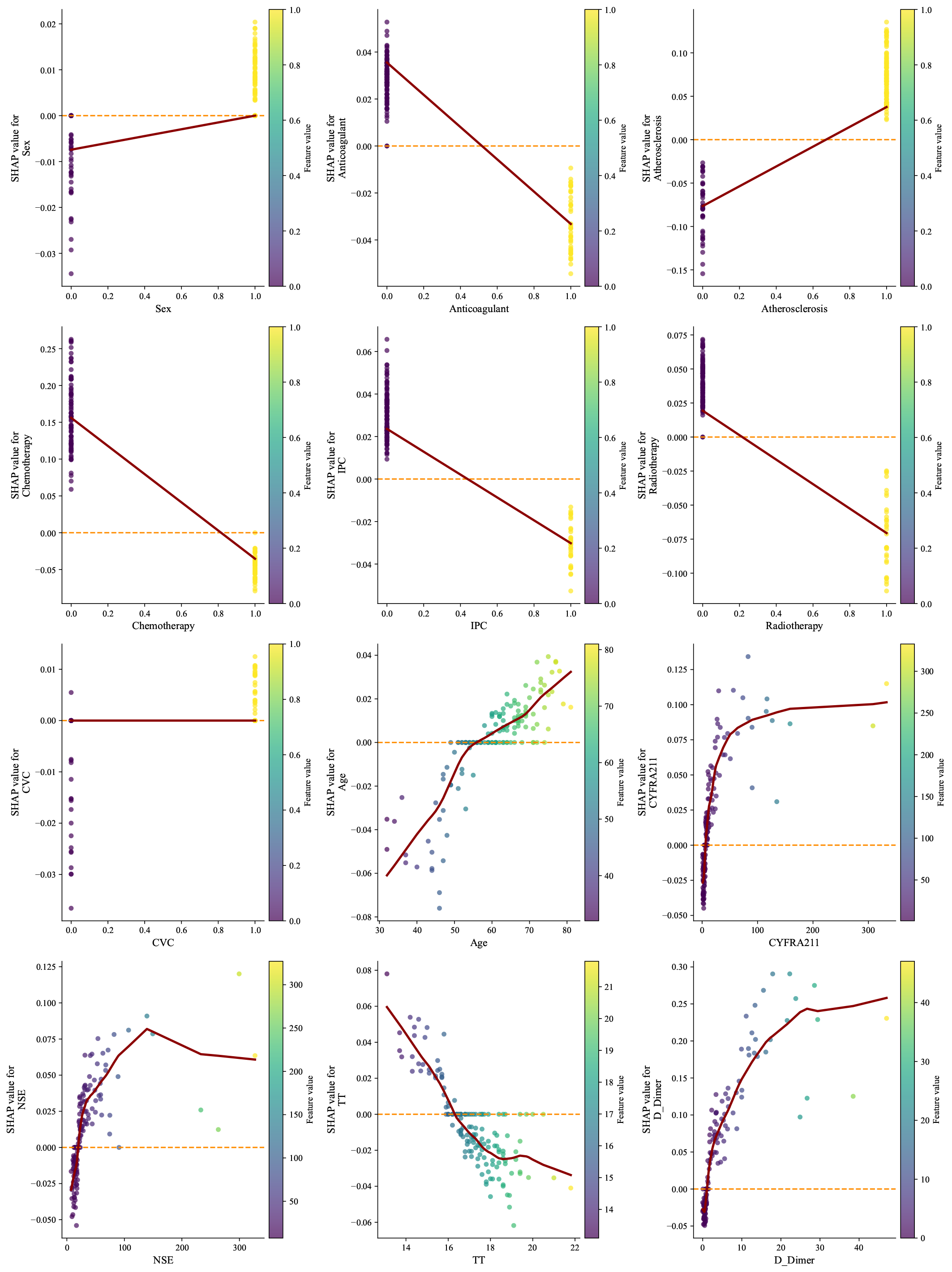


Supplementary Figure 2. SHAP dependence plots for the 12 predictors included in the final ANN model. Each plot illustrates the relationship between an individual predictor and its corresponding SHAP value, showing how changes in predictor values influence the predicted risk of VTE.
